# Dynamics of oral microbiome acquisition in healthy infants: A pilot study

**DOI:** 10.3389/froh.2023.1152601

**Published:** 2023-03-30

**Authors:** Yihong Li, Prakaimuk Saraithong, Lanxin Zhang, Ashley Dills, Bruce J. Paster, Jin Xiao, Tong Tong Wu, Zachary Jones

**Affiliations:** ^1^ Master of Public Health Program, Department of Public and Ecosystem Health, Cornell University, Ithaca, NY, United States; ^2^ Department of Internal Medicine, Medical School University of Michigan, Ann Arbor, MI, United States; ^3^ Department of Molecular and Cell Biology, University of California Berkeley, Oakland, CA, United States; ^4^ Family Translational Research Group, New York University College of Dentistry, New York, NY, United States; ^5^ Molecular Microbiology & Genetics, The Forsyth Institute, Cambridge, MA, United States; ^6^ Department of Oral Medicine, Infection, and Immunity, Harvard School of Dental Medicine, Boston, MA, United States; ^7^ Eastman Institute for Oral Health, University of Rochester Medical Center, Rochester, NY, United States; ^8^ Department of Biostatistics and Computational Biology, University of Rochester Medical Center, Rochester, NY, United States; ^9^ Department of Basic Science and Craniofacial Biology, New York University College of Dentistry, New York, NY, United States

**Keywords:** oral microbiota, microbial diversity, infant-mother dyads, postpartum, microbial initial acquisition

## Abstract

**Objectives:**

The human oral microbiota is one of the most complex bacterial communities in the human body. However, how newborns initially acquire these bacteria remains largely unknown. In this study, we examined the dynamics of oral microbial communities in healthy infants and investigated the influence of the maternal oral microbiota on the acquisition of the infant's oral microbiota. We hypothesized that the infant oral microbial diversity increases with age.

**Methods:**

One hundred and sixteen whole-salivary samples were collected from 32 healthy infants and their biological mothers during postpartum and 9- and 15-month well-infant visits. Bacterial genomic DNA was extracted and sequenced by Human Oral Microbe Identification using Next Generation Sequencing (HOMI*NGS*) methods. The Shannon index was used to measure the microbial diversity of the infant-mother dyads (alpha diversity). The microbial diversity between the mother-infant dyads (beta-diversity) was calculated using the weighted non-phylogenetic Bray-Curtis distance in QIIME 1.9.1. Core microbiome analysis was performed using MicrobiomeAnalyst software. Linear discriminant analysis coupled with effect size analysis was used to identify differentially abundant features between mother and infant dyads.

**Results:**

A total of 6,870,571 16S rRNA reads were generated from paired mother–infant saliva samples. Overall, oral microbial profiles significantly differed between the mother and infant groups (*p* < 0.001). The diversity of the salivary microbiomes in the infants increased in an age-dependent manner, whereas the core microbiome of the mothers remained relatively stable during the study period. Breastfeeding and gender did not affect the microbial diversity in infants. Moreover, infants had a greater relative abundance of Firmicutes and a lower abundance of Actinobacteria, Bacteroidetes, Fusobacteria, and Proteobacteria than their mothers. The SparCC correlation analysis demonstrated constant changes in infants' oral microbial community network (*p* < 0.05).

**Conclusions:**

This study provides new evidence that the oral cavities of infants are colonized by a distinct group of bacterial species at birth. The acquisition and diversity of changes in oral microbial composition are dynamic during the first year of an infant's life. Before reaching the second birthday, the composition of the oral microbial community could be more similar to that of their biological mothers.

## 1. Introduction

The human oral microbiota is one of the most complex bacterial communities in the human body, with over 700 bacterial species having been identified in the oral cavity (1, 2). However, the initial acquisition and development of complex oral microbiota in human infancy have not been fully delineated. Moreover, the factors influencing the colonization and maturation of the oral microbiome during the first year of an infant's life also remain uncharacterized. Further, the significance of an early establishment of oral microbiota to our future health remains unclear.

While the oral microbiome, in general, influences human growth and the development of the immune system, there is some debate as to when the infant or fetus is first exposed to bacteria. The fetus develops in a sterile state under normal conditions. However, recent studies using advanced molecular approaches have detected bacterial DNA in the placenta tissue and amniotic fluid in healthy pregnancies (3). The placenta harbors a unique microbiome that is more similar to flora in the oral community compared to other human body sites, such as the skin, nasal, vaginal, and gut microbiomes (4, 5). The early colonization of the oral microbiota in infants, primarily members of the indigenous biota, is acquired at birth or begins postpartum (6). Recent developments in the human microbiome suggest that placental microbial inflammation may contribute to an increased risk of preterm birth (7, 8). Previous studies have reported that the newborn oral cavity is rapidly dominated by Bifidobacterium species, specifically *Streptococcus*, *Gemella*, *Veillonella*, *Granulicatella*, and *Rothia*, followed by *Haemophilus*, *Actinomyces*, *Porphyromonas*, *Prevotella*, and *Neisseria* genera, immediately after birth (6, 9, 10). Their presence might play a vital role in host defense, not only in excluding potential exogenous pathogens but also as stimuli for the development of the immune system in infants (11, 12). Several factors can influence the acquisition and establishment of the oral microbiota in infants, such as maternal microbiota, mode of delivery, feeding practice, diet, caretaker contacts, and antibiotic use (10, 13–18).

In this study, we examined the dynamics of oral microbial communities in healthy infants from postpartum to 15 months of age and investigated time-correlated changes in microbial composition between infants and their mothers. We used culture-dependent methods for *Streptococcus mutans* detection and quantification and 16S rRNA-based human oral microbe identification using next-generation sequencing (HOMI*NGS*) on saliva samples in this study. We hypothesized that human newborns acquire a distinct group of bacteria at birth which is significantly different from their biological mothers. The oral microbial diversity increases with age and is influenced by the mother's oral microbiota.

## 2. Materials and methods

### 2.1. Study population

This pilot study was concurrent with an ongoing research project targeting couple and parent-child coercion to improve health behaviors [ClinicalTrials.gov Identifier: NCT03163082] (19). Thirty-two families were randomly selected from a pool of participants. Information on the sample size estimation is included in Supplementary Tables S1, S2 and Figure S1. The families were followed up from the children's birth for 15 months. Complete assessments included an administrated questionnaire survey on family functioning, oral health behaviors, and feeding practices at baseline (postpartum) and nine and 15 months of age. Follow-up well-infant visits were conducted at the Bellevue Hospital Pediatric Clinic, Gouverneur Hospital Pediatric Clinic, and New York University College of Dentistry. The recruitment procedure and characteristics of the families have been previously described in detail (20).

### 2.2. Ethics statement

The study involving human participants was reviewed and approved by the Institutional Review Boards of the New York University School of Medicine, New York University College of Dentistry (Research Proposal Oversight Committee), and the New York City Health and Hospital Corporation (for the Bellevue Hospital Center) for human subjects participating in research activities. All parents provided informed consent and permitted their children to participate in the study.

### 2.3. Saliva *Streptococci mutans* assessment

Whole saliva samples were collected from the mothers and infants at each visit. After resting for 5 min without talking, the mothers were asked to rinse their mouths with sterile water, chew on a piece of paraffin wax for 30 s, and expectorate directly into a graduated 50 ml sample collection tube on ice. For the infants, sample collection was performed using a sterile cotton swab to swab the infant's mouth around the dental ridges and rotated for 10–30 s until the swab was saturated. The swab was immediately placed into a pre-labeled sterile vial containing 2 ml of pre-reduced reduced transport fluid [RTF (21)]. The swab tip was broken off, and the lid was screwed on tightly. All saliva samples were immediately transferred on ice to a microbiology laboratory (New York University College of Dentistry). Ten 10-fold serial dilutions (10^−1^ to 10^−3^) of the sample were used to obtain accurate colony-forming unit (CFU) reads. Diluted samples (50 µl) were plated on mitis salivarius agar with potassium tellurite-bacitracin plates (MSB, Difco Laboratories Inc., Detroit, MI, Unitted States) using an Autoplate Spiral Plating System (Advanced Instruments, Inc., Norwood, MA, United States). After a 72 h anaerobic incubation (85% N2, 10% CO2, and 5% H2) at 37°C, the number of CFU of *S. mutans* was assessed and recorded.

### 2.4. Bacterial genomic DNA extraction

Total salivary bacterial genomic DNA was extracted from 1 ml of whole saliva samples (mothers) or swab samples (infants) using a modified DNA purification kit (Epicenter, Madison, WI, United States) as previously described (22). An additional 10 µl proteinase K (10 mg/ml in TES buffer-10 mM Tris–HCl, pH 8.0; 1 mM EDTA; 100 mM NaCl), 10 µl lysozyme stock solution (100 mg/ml in TES buffer), and 2 µl mutanolysin (5,000 U/ml in PBS) were added to the sample followed by a phenol/chloroform/isoamyl alcohol extraction procedure. Final DNA quality and concentration were measured using a NanoDrop 1000 spectrophotometer (Thermo Scientific, Wilmington, DE, United States). The bacterial DNA was stored at −20°C.

### 2.5. 16s rDNA sequencing and data processing

The bacterial genomic DNA 16S V1-V3 libraries were prepared and sequenced using the HOMI*NGS* assay (Forsyth Institute Sequencing Facility, Cambridge, MA, United States) according to a modified protocol as previously described (23, 24). Briefly, 10–50 ng of bacterial DNA was PCR-amplified using V3-V4 forward (341F) (5′-AATGATACGGCGACCACCG AGATCTACACTATGGTAATTGTCCTACGGGAGGCAGCAG-3′) and reverse (806R) (5′-CAAGCAGAAGACGGCATACGAGATNNNNNNNNNNNNAGTCAGTCAGCCGGACTACHVGGGTWTCTAAT-3′) primers, and then purified using AMPure beads. A 12 pM denatured library mixture, spiked with 20% PhiX library, was sequenced using the MiSeq System (Illumina, San Diego, CA, United States).

An average of >50,000 sequences (441 bp per sequence) were obtained for each sample. Samples with reads less than 2,000 bp and chimeric sequences were removed from further analysis (Supplementary Figure S2). Quality control was done using the Quantitative Insights into Microbial Ecology “split_libraries.py” script (QIIME 1.91) program with the default quality control cut-offs for sequence lengths (minimal 200), end-trimming with a minimum quality score of 25. The paired reads were then merged with the “join_paired_ends.py” script (QIIME 1.91) (25). Bacterial identification was based on 660 species-specific oligonucleotide probes designed for the Human Oral Microbiome Database (HOMD) developed at the Forsyth Institute (https://homings.forsyth.org/index2.html). An additional panel of 107 genus-specific probes was used in the analysis. The identification was done using in-house software called ProbeSeq, which searches for the exact match in each quality-filtered and merged read (23). Sequencing data that passed the quality controls were included in this study and assigned to its open-reference operational taxonomic unit (OTU). OTUs selected for downstream analysis were those with at least 20% of their value containing at least four counts and with at least 10% of variances (measured by inter-quantile range). The OTU table of raw counts was normalized to an OTU table of relative abundance at the phylum, class, order, family, genus, and species levels (23).

### 2.6. Statistical analysis

The relative abundances of bacterial taxa were compared between the mother and infant groups, visits, sex, and breastfeeding practices. The Shannon index was used to measure alpha diversity using the phyloseq package from the R vegan package (26). The results are displayed as boxplots containing multiple groups based on experimental factors. Statistical differences were assessed using the Wilcoxon–Mann–Whitney and Kruskal–Wallis tests. Beta diversity was measured by calculating the non-phylogenetic Bray-Curtis distance computed using the QIIME workflow (25) for phylogenetic differences. A heat plot was generated to highlight the major genera driving the clustering of samples from the mothers and infants at different visits.

Ordination-based principal coordinates analysis (PCoA) was performed on each beta diversity metric to generate two-dimensional plots to highlight the separation of infants from mothers based on time points, genders, and feeding practices. Differences in oral microbial composition between the groups were tested using permutational multivariate analysis of variance (PERMANOVA) on beta diversity matrices, adjusting for sequencing results.

The relative abundances at the phylum, genus, and species levels were plotted to visualize the differences in microbial composition between mothers and infants, and between breastfed and non-breastfed infants. The species-level relative abundance was compared between the time points among infants and their mothers using MaAsLin2 (27). Core microbiome analysis was performed using MicrobiomeAnalyst (28, 29). The results are shown as heatmaps containing taxa detected in over 20% of the population, with a relative abundance above 0.01%. The y-axis represents the prevalence of the taxa given the detection threshold [relative abundance (%)] on the x-axis. Heatmaps were used to compare the core taxa of mothers and infants in the postpartum, 9-month, and 15-month visits. Linear discriminant analysis (LDA) coupled with effect size (LEfSe) analysis (30) and nonparametric factorial Kruskal–Wallis sum-rank test were used to identify differentially abundant features between mothers and infants. The difference was considered significant if the *p*-value (FDR-adjusted) was less than 0.1 and the Log LDA score was over 2.0. The results were organized into plots where the identified taxa were labeled along the y-axis with the LDA score on the x-axis. A higher LDA score indicated that mothers had a higher relative taxon abundance. SparCC correlation analysis was used to investigate infant oral microbiota's evolution and reveal the relationship between microbial communities. The correlation threshold was set at 0.5, *p* < 0.05. Correlated taxa were connected by lines in the plots.

In addition to microbial alpha and beta diversities, the *S. mutans* CFU data obtained from the MSB plates was transformed to logarithm (log10) values. The nonparametric Wilcoxon-Mann-Whitney test and Kruskal–Wallis one-way analysis of variance were used for mean comparisons between mothers and infants, genders, and visits. Statistical analysis was conducted using the Stata Statistics program (version 17.0; StataCorp LLC, College Station, TX, United States).

All statistical tests were two-sided, with a *p-*value of less than 0.05, denoting statistical significance. The false discovery rate (FDR) adjusted with Benjamini–Hochberg (*q* < 0.05) was considered statistically significant.

## 3. Results

The baseline visit (postpartum) involved 32 mother-infant dyad participants. The age of the mothers ranged from 18 to 34 years, with a mean value of 24.2 ± 4.2. All infants were born full term; 22 were male and ten were female. The infant's race breakdown was as follows: 15 Latino, five African American, three Asian, and nine mixed races. At the 9-month and 15-month visits, 13 mother-infant dyads completed the follow-up examinations (Supplementary Figure S3). A total of 116 saliva samples were collected and processed for culture-dependent evaluations of *S. mutans* colonization, bacterial 16S rDNA sequences, and HOMI*NGS* data analysis.

### 3.1. *S. mutans* colonization

Based on the cultivation results, *S. mutans* was detected in 96.8% of salivary samples from the mothers at the baseline visit and 100% at the follow-up visits. The mean log_10_ value of *S. mutans* ranged from 4.67 ± 1.23 at the postpartum visit to 4.88 ± 0.99 15 months later. None of the infants were *S. mutans* positive at birth. Only two infants (15.4%) were positive at 9 months (mean = 1.84 ± 0.34, log_10_ value) and 15 months (mean = 3.47 ± 1.03, log_10_ value) (Table 1). *S. mutans* colonization was not influenced by the gender, age, or breastfeeding experience of the infants. Based on the 16S rDNA sequences and HOMI*NGS* data analysis, the top 20 predominant genera/species constituted approximately 86.0% and 88.6% of the sequences detected in the saliva samples from newborns and mothers, respectively. As shown in Table 2, the *Streptococcus* genus was the major component of the infant salivary microbiota, comprising more than 72.5% of the total identified bacterial genera. At the species level, *S. mutans* was detected in 0.0088% of the newborn saliva, along with *S. sanguinis* and other taxa of oral *Streptococcus* species (Table 3).

**Table 1:** Colonization of *S. mutans* in the saliva of the mother-infant dyads.

Visit	Postpartum		9th month		15th month	
	*N* = 32		*N* = 13		*N* = 13	
	Positive (%)	Mean* ± SD	Positive (%)	Mean* ± SD	Positive (%)	Mean* 2 SD
Infant	0	0	2 (15.4%)	1.84 ± 0.34	2 (15.4%)	3.47 ± 1.03
Mother	31 (96.8%)	4.67 ± 1.23	13 (100%)	4.80 ± 1.11	13 (100%)	4.88 ± 0.99

* Mean was calculated based on the colony-forming units obtained from the MSB-selective medium and log10 transferred.

**Table 2:** Comparison of the percentage of top 20 16S rDNA genus probes present in the saliva of the mother-infant dyads.

Probe ID	Genus/Specie Name	Group	
		Infant	Mother
GP-081	Streptococcus_Genus_probe_4	71.77%	39.58%
RO-03	*Rothia_mucilaginosa*	10.08%	24.26%
PR-14	*Prevotella_melaninogenica*	0.30%	3.62%
GP-110	Granulicatella_Genus_probe	0.38%	3.00%
ST-20	*Streptococcus_sanguinis*	0.11%	1.82%
GE-04	*Gemella_sanguinis*	0.02%	1.64%
GP-126	Streptococcus_Genus_probe_1	0.14%	1.61%
GE-02	*Gemella_haemolysans*	4.43%	1.32%
PR-09	*Prevotella_histicola*	0.06%	1.19%
GP-060	Neisseria_Genus_probe_2	0.07%	1.17%
GP-004	Actinomyces_Genus_probe_4	0.12%	1.15%
GP-073	Rothia_Genus_probe	0.26%	0.99%
HA-05	*Haemophilus_parainfluenzae*	0.19%	0.91%
GP-089	Veillonella_Genus_probe_2	0.44%	0.63%
PO-09	Porphyromonas_sp_oral_taxon_279	0.14%	0.60%
RO-02	*Rothia_dentocariosa*	0.01%	0.59%
OR-01	*Oribacterium_sinus*	0.01%	0.55%
SO-01	*Solobacterium_moorei*	0.04%	0.47%
FU-10	*Fusobacterium_periodonticum*	0.07%	0.47%
GP-063	Parvimonas_Genus_probe	0.01%	0.43%

**Table 3:** Comparison of the percentage of top genus and species of *Streptococcus* 16S rDNA probes present in the saliva of the mother-infant dyads.

Probe ID	Genus/Specie Name	Group	
		Infant	Mother
ST-09	*Streptococcus_anginosus*	0.0036%	0.0986%
ST-10	*Streptococcus_constellatus*	0.0032%	0.1042%
ST-11	*Streptococcus_cristatus*	0.0031%	0.0016%
ST-12	*Streptococcus_downei*	0.0000%	0.0004%
ST-14	*Streptococcus_intermedius*	0.0023%	0.0634%
ST-15	*Streptococcus_mutans*	0.0088%	0.0782%
ST-16	*Streptococcus_parasanguinis_II*	0.0789%	0.0448%
ST-20	*Streptococcus_sanguinis*	0.1148%	1.8175%
ST-21	*Streptococcus_sobrinus*	0.0007%	0.0671%
ST-22	*Streptococcus_sp*_oral_taxon_064	0.1317%	0.0785%
ST-23	*Streptococcus_sp*_oral_taxon_066	0.0707%	0.0385%
ST-24	*Streptococcus_sp*_oral_taxon_068	0.0064%	0.0063%
ST-26	*Streptococcus_sp*_oral_taxon_431	0.1049%	0.0535%
ST-27	*Streptococcus_sp*_oral_taxon_486	0.0256%	0.0152%
ST-28	*Streptococcus_sp*_oral_taxon_487	0.0000%	0.0004%
GP-126	Streptococcus_Genus_probe_1	0.1411%	1.6107%
GP-127	Streptococcus_Genus_probe_2	0.0039%	0.0024%
GP-128	Streptococcus_Genus_probe_3	0.0411%	0.0269%
GP-081	Streptococcus_Genus_probe_4	71.7651%	39.5773%

### 3.2. Comparison of microbiome diversity between mothers and infants

MiSeq sequencing, obtained from 116 clean bacterial genomic DNA samples, yielded 6,870,571 reads with a mean read length of 460 bp (330–591 bp). On average, there were 65,270 reads per sample (range 120 to 151,900, median = 47,330). Four samples that produced less than 1,500 bp were eliminated from the final data analysis. Among the 767 probes tested, 600 (78.2%) tested positive, 484 (63.5%) in the infant samples and 561 (73.1%) in the mother samples. Among those reads, 3,819,592 (55.6%) matched uniquely with one genus probe, 2,088,913 (30.4%) matched uniquely with one species probe, and 960,108 (14.0%) were unmatched.

**Figure 1:**
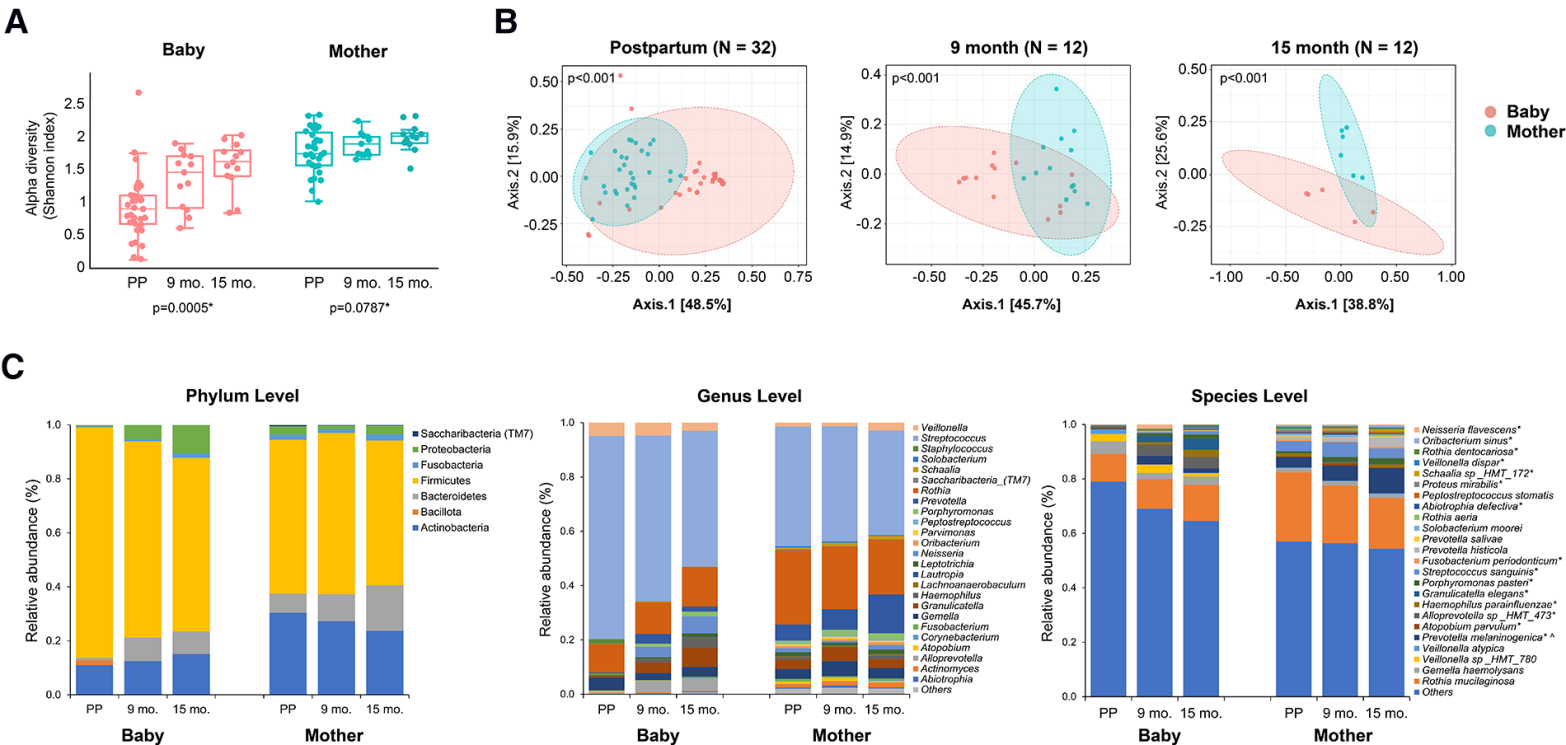
Comparison of the oral microbial alpha diversity between the infant and mother groups. (**A**) The figure shows a significant difference in the alpha diversity measured by the Shannon index among the three visits in the infant group. *The Kruskal–Wallis one-way analysis of variance. (**B**) Beta diversity, measured by the Bray-Curtis Index, of mothers and infants. A significant difference was detected between mothers and infants at all three-time points (postpartum, 9 months, and 15 months) for the Shannon index and Bray-Curtis index. (*p* < 0.001, Wilcoxon–Mann–Whitney test). (**C**) Relative abundance at the phylum, genus, and species levels. The top 25 most abundant genera and species are plotted. * and ^ indicate that the relative abundance of taxa is significantly different across times in infants and mothers, separately, with adjusted *p*-value <0.05.

The overall oral microbial community profiles and abundance of taxa and genera were markedly different between the infant and mother groups (Wilcoxon test; *p* < 0.001). Mothers had a more diverse salivary community than their infants at postpartum, 9 months, and 15 months, as measured by the alpha diversity Shannon index (Figure 1A, Kruskal–Wallis one-way analysis of variance, *p* < 0.001). The salivary microbiome diversity in infants increased at 9 and 15 months compared to that in the postpartum period. The alpha diversity of the salivary microbiomes of mothers was relatively stable over time. As the principal coordinate analysis (PCOA) plot illustrated, the beta diversity (Bray-Curtis Index) between the infants and mothers was significantly different at all three visits (Figure 1B). The microbial communities in mothers showed less variability than those of the infants, as indicated by the smaller range of sample distribution in the plots.

### 3.3. Comparison of core oral microbiome between mothers and infants

The relative abundances of the salivary microbiome at the phylum, genus, and species levels are shown in Figure 1C. Overall, the infant group had a higher relative proportion of Firmicutes (*p* < 0.05) and fewer Actinobacteria, Bacteroidetes, Fusobacteria, and Saccharibacteria (*p* < 0.001) compared to those in the mothers. Among the top 25 most abundant genera and species, significant changes in abundance over time were found in the following in infants: *Neisseria flavescens, Oribacterium sinus, Rothia dentoscriosa, Veillonella dispar, Schaalia* sp_HMT_172*, Proteus mirabilis, Abiotrophia defective, Rothia aeria, Fusobacterium periodonticum, Streptococcus sanguinins, Porphyromonas pasteri, Granulicatella elegans, Haemphilus parainfluenza, Alloprevotella* sp_HMT_473*, Atopobium parvulum,* and *Prevotella melaninogenica*. The change in the relative abundance of *Prevotella melaninogenica* was also significant in the mothers during the visits.

**Figure 2:**
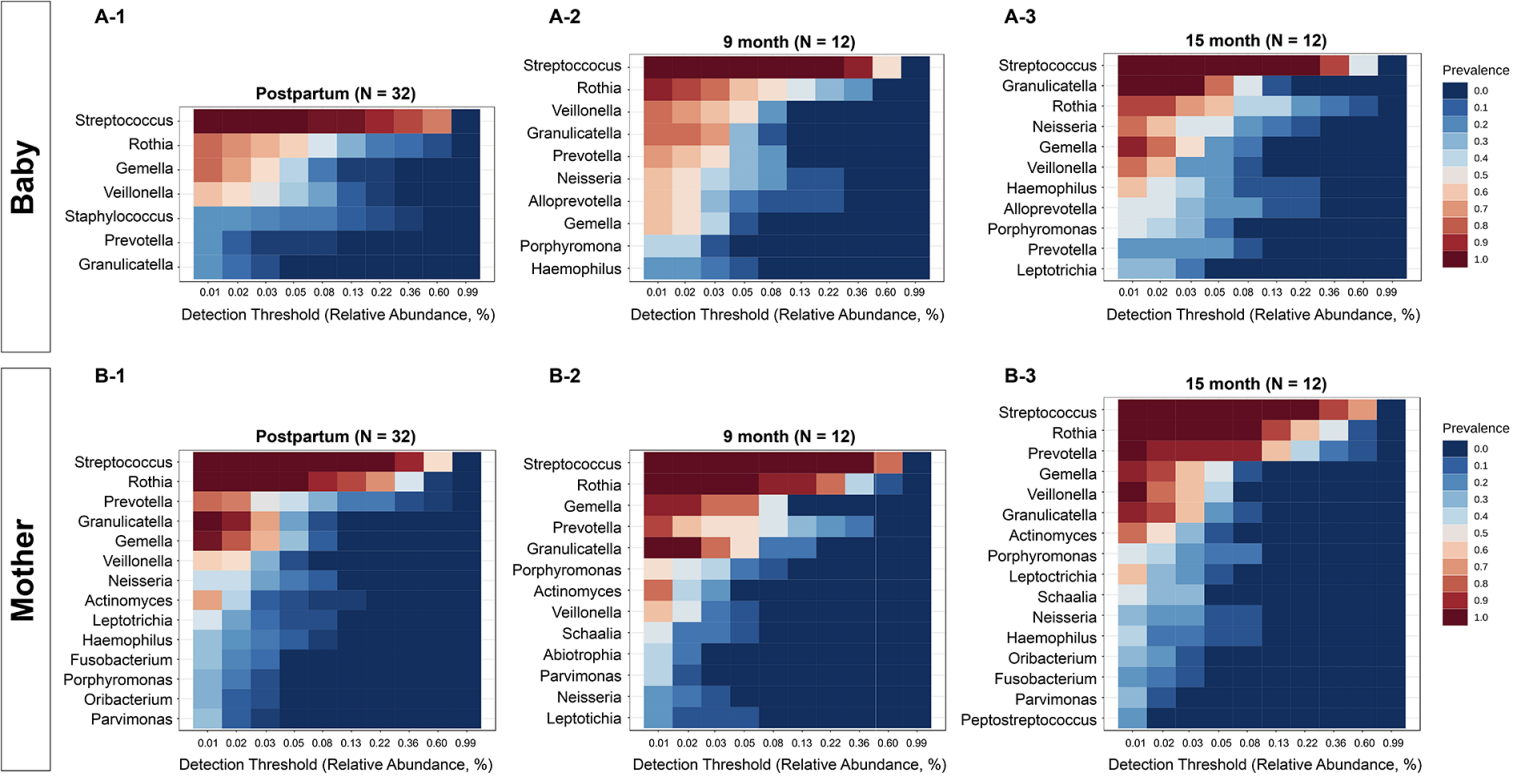
Comparison of core oral microbiome between mothers and infants. The core genera for infants (**A**) and mothers (**B**) were defined with 20% sample prevalence and 0.01% relative abundance. The core microbiome of the mothers was relatively stable, while the core oral microbiome in infants evolved over time. For infants, *Neisseria*, *Alloprevotella*, and *Haemophilus* gained abundance after 9 months.

The core genera for the infant group (Figure 2A) and mother group (Figure 2B) were defined as having 20% sample prevalence and 0.01% relative abundance at postpartum (A-1 and B-1), 9 months (A-2 and B-2) and 15 months (A-3 and B-3). The core oral microbiome of mothers was relatively stable during the study period, while the core oral microbiome in infants evolved over time. Similar to salivary microbial diversity, the compositions were relatively stable for the mothers during the 15 months. In contrast, the salivary microbiome of children started with fewer abundant taxa at the initial visit and was enriched with more taxa at 9 and 15 months. For example, *Streptococcus, Rothia, Prevotella, Gemella, Veillonella, Neisseria,* and *Actinomyces* were constant taxa at different time points in mothers. For infants, *Prevotella*, *Neisseria*, *Alloprevotella*, and *Haemophilus* gained abundance after 9 months. The overall differences in diversity between infants and mothers decreased at the 15-month visit.

### 3.4. Discriminating features between mothers and infants

**Figure 3:**
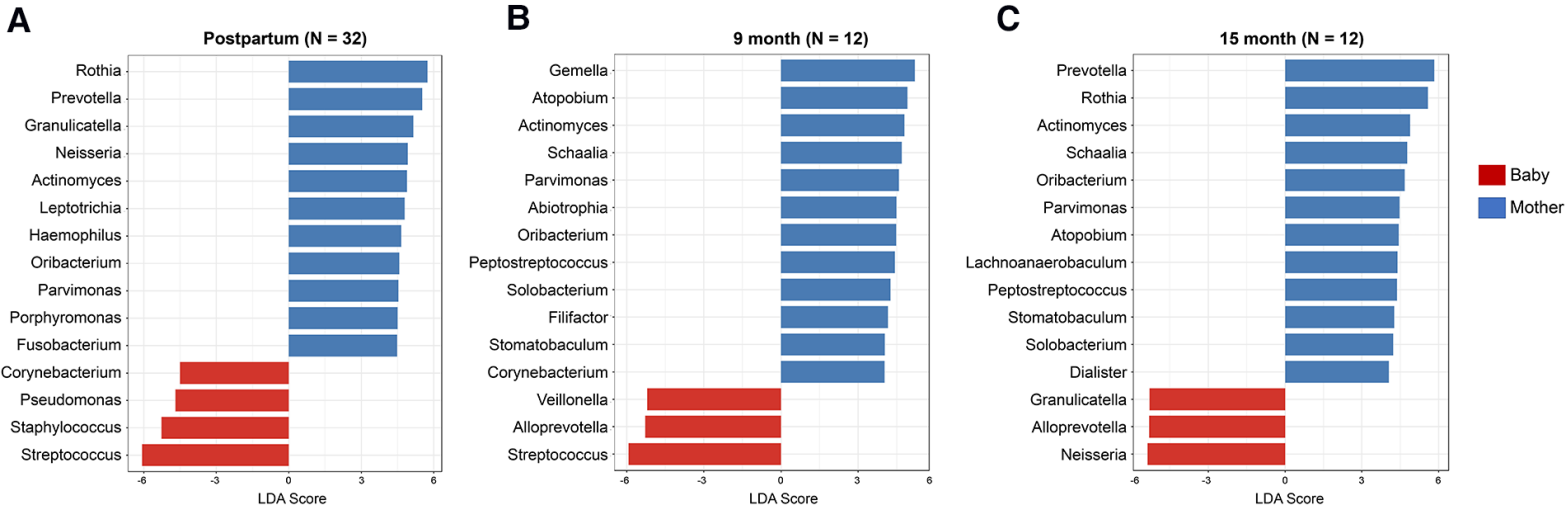
Differentially abundant oral microorganisms between mothers and infants in early life. The genera with significant differences between the mothers and the infants were detected by Linear discriminant analysis effect size test (LEfSe, FDR 0.05, LDA 2.0) using Kruskal–Wallis test for the three visits (**A**) postpartum, (**B**) 9 months, and (**C**) 15 months. Among the genera with a significant difference, mothers have a higher number of genera with higher abundance. The differences in *Corynebacterium* and *Staphylococcus* between the mothers and the infants became less significant from postpartum to 15 months of age.

The genera with significant differences between mothers and infants were detected by linear discriminant analysis effect size test (LEfSe, FDR 0.05, LDA 2.0) and compared at the postpartum (Figure 3A), 9-month (Figure 3B) and 15-month visits (Figure 3C). The mothers had a higher number of genera with a significantly higher abundance. *Streptococcus* and *Staphylococcus* genera were more abundant in the early life of infants than in their mothers. However, this difference was not observed after 15 months. In contrast, *Corynebacterium* had a higher abundance in infants at the postpartum visit but a reversed abundance in mothers at 9 months. Still, there was no difference between mothers and infants at the 15th-month visit (Figure 3C).

### 3.5. Correlation analysis of oral microbial development in infants

**Figure 4:**
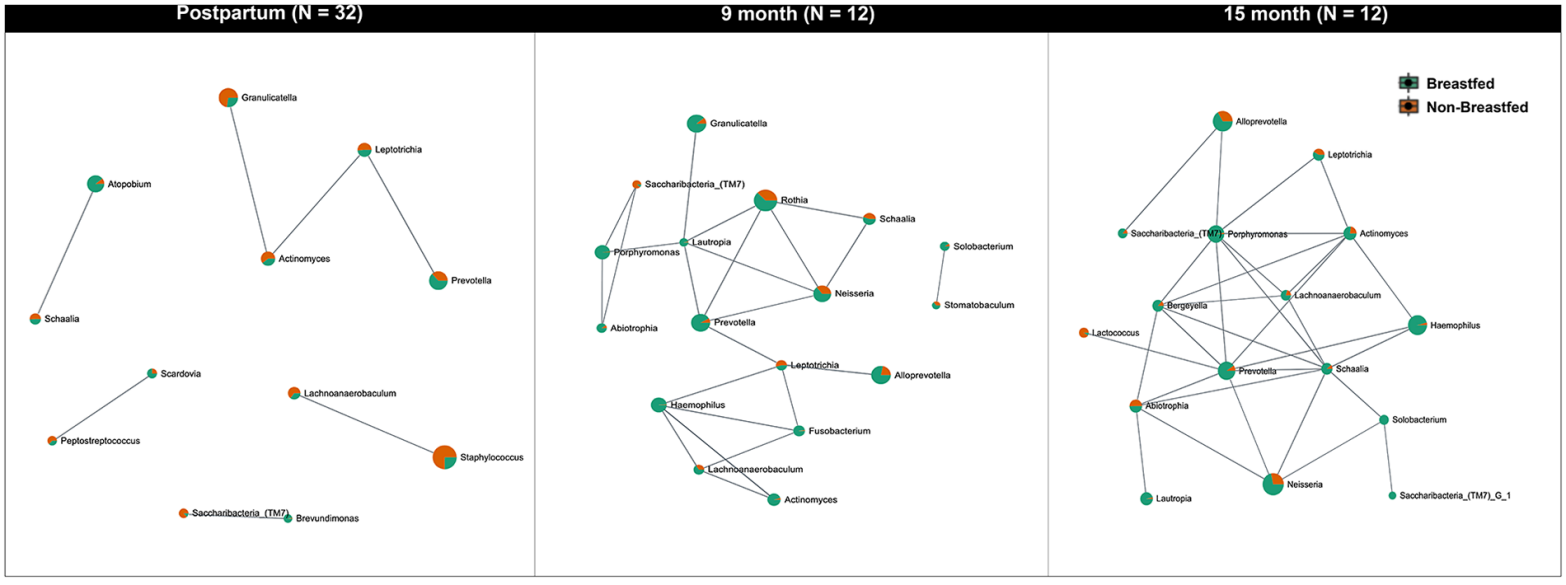
Network analysis of the oral microbiota evolution in infants. The figure indicates the network of microbial communities at the genus level built from SparCC correlation coefficients stratified by feeding methods. The nodes represent genera of bacteria. The edges between the nodes represent the correlation coefficients between genera. The results show that the relationship between the oral microbiomes in infants is constantly changing and that more genera begin to be interconnected over time. The correlation threshold is set at 0.5, *p* < 0.05.

While comparing the relative abundance and diversity of oral microbes within each group over time, we found that the microbiome diversity in infants significantly increased after birth (Kruskal–Wallis test, *p* < 0.001). SparCC correlation analysis demonstrated a network of salivary genera (Figure 4). The network was further stratified by the feeding method. The correlation threshold between genera was set at 0.5 with a *p*-value of less than 0.05. Significantly, the salivary microorganism network evolved in early life. At the postpartum visit, Prevotella was highly associated with Leptotrichia, Actinomyces, and Granulicatella (Figure 4A). More members joined this connected network at nine months, including *Lautropia, Schaalia, Neisseria, Abiotrophia, Porphyromonas*, and *Saccharibacteria* (TM7) (Figure 4B). At 15 months of age, *Lactobacillus* was also connected to the network. (Figure 4C).

We also examined the effects of gender and feeding experience on microbial diversity in infants. The influence of feeding methods (breastfed vs. milk-fed) on the early life (postpartum) salivary microbiome in infants was further examined by assessing alpha diversity (Shannon index, Supplementary Figure S4A) and beta diversity (Bray-Curtis index, Supplementary Figure S4B). The observed differences in microbial composition (Supplementary Figure S4C) between breastfed and non-breastfed infants were not statistically significant.

### 3.6. Stability of maternal oral microbiome

We further assessed the stability of the maternal oral microbiome among mothers who completed all three visits (*N* = 8). The alpha diversity was evaluated by the Shannon index (Supplementary Figure S5A1), which showed that mothers' salivary microbial diversity at postpartum was significantly less than that at 9 months (*p* = 0.02) and 15 months (*p* = 0.001). However, the Chao1 index indicated no significant alteration in the microbial diversity of the mothers during the 15 months (*p* > 0.05). The salivary microbiome diversity in mothers remained relatively stable (Supplementary Figure S5A2). The beta diversity measured by the Bray-Curtis index demonstrated no significant change across time (Supplementary Figure S5B). We also explored the stability of the infant oral microbiome and comparing that with their mothers. The results revealed consistent differences between the mother-infant paired samples at all three-time points (Supplementary Figure S6).

## 4. Discussion

The rationale for including the *S. mutans* assessment was threefold: (1) to determine the initial *S. mutans* acquisition in a healthy infant cohort; (2) to compare the traditional culture methods with new molecular 16S rRNA sequencing methods in determining *S. mutans* present in the infant's saliva; and (3) to explore the inter- and intra-microbial composition correlation between *S. mutans* and other microbial species, especially at an early establishment stage. In this study, we examined the initial acquisition and diversity of the oral microbiomes of 32 healthy infants at three time points (postpartum, 9 months, and 15 months of age) and evaluated the changes in their microbiomes. Particular attention was paid to exploring the factors influencing the establishment of and dynamic changes in the oral microbiome of infants. *S. mutans* is the principal etiological agent of dental caries (31, 32). Early colonization is significantly correlated with an increased risk of the disease (32–34). Thus, the first objective of this study was to examine the time of initial colonization of *S. mutans* in newborns. As expected, using the conventional cultivation method, we observed that *S. mutans* could be detected in 96.8% to 100% of the saliva in mothers at each visit. *S. mutans* was not detected in infants' saliva at birth but in 15.4% of infants at 9 and 15 months of age. These results are consistent with those of previous studies (34–36). Several studies have reported a higher percentage of children under 14 months of age colonized with *S. mutans* (37, 38). Some studies have suggested that the *S. mutans* initial colonization in the oral cavity can vary between 7 and 36 months, coinciding with the eruption of the primary teeth (39–41). Since only 32 infants were examined in the study and only two were *S. mutans* positive, the study was unable to conclude the correlation of *S. mutans* colonization in the mother-infant dyads.

Based on the HOMI*NGS* assay, *S. mutans* was detected in the infant saliva in very low numbers, along with *S. sanguinis* and other taxa of oral *Streptococcus* species. The results confirmed that *S. mutans* may be one of the early colonizers in infant saliva and a critical facilitator for establishing a *Streptococcus*-associated oral polymicrobial community (42). Furthermore, the core genera in the newborn saliva included not only *Streptococcus* but also *Rothia*, *Gemella*, *Veillonella*, *Staphylococcus*, *Prevotella*, and *Granulicatella*. The findings are similar to those of previous studies (43) and further support the caries ecological plaque hypothesis (44), suggesting that the early acquisition of the diverse microbiota may dictate the microbial community establishment in the saliva and oral health of an individual.

We further assessed the oral microbiome diversity in infants and relative abundance at postpartum, 9 months, and 15 months of age and compared them to their mothers. We found that, at the genus level, mothers had a more diverse microbial community than their infants. The mothers' salivary microbiome profile, diversity, and core composition remained relatively stable over time. In contrast, the alpha diversity of the bacterial community increased significantly with age in the infants. These findings are consistent with those of a recent study by Ramadugu et al., which demonstrated that the salivary microbial diversity and richness in infants increased with infant age, regardless of the oral health status, education, delivery route, and breastfeeding practice of mothers (45).

Although beta diversity was significantly different between the mother-infant dyads at each time point, we observed that 85.7% (postpartum), 80.0% (9 months), and 90.1% (15 months) of the core genera in the saliva from infants could be identified in the core microbiome of mothers. More genera were connected at 9 and 15 months, and more correlation networks were detected in the infant saliva, suggesting that the microbial community in saliva constantly changed over time. The results indicated that the oral cavities of infants were colonized by a distinct group of bacterial species at birth. As more bacteria colonized the oral cavity in infants, the diversity discordance between infants and their mothers decreased. It seems possible to speculate that the overall composition of the oral microbial community in infants could be similar to that of the mothers before reaching their second birthday. A recent study reported that the salivary microbiomes of infants become more adult-like with age (45). However, Ramadugu's analysis could not determine the bacterial sources in infants. On the other hand, Ferrett et al. have demonstrated mother-to-infant microbiome transmission routes that influence the development of the infant microbiome based on genomic identification, strain-level metagenomic profiling (single-nucleotide variant profiling), and longitudinal sampling approaches (43).

The mechanisms underlying infants' initial acquisition and subsequent colonization of the oral microbiome remain unclear. It was hypothesized that oral microbiome diversity in infants increases with age. In this study, we observed that 74.8% of the oral microbiome belongs to the *Streptococcus* genus in saliva during the first month of an infant's life. The percentage decreased to 60.9% at 9 months and 50.0% at 15 months. The second most abundant genus was *Rothia* which increased from 10.5% during the postpartum period to 11.7% and 14.5% at 9 and 15 months, respectively. The genus *Neisseria* increased 15.8 times in 9 months and 77 times in 15 months. The genera *Alloprevotella*, *Granulicatella*, *Prevotella*, and *Haemophilus* also increased significantly during the first 15 months of life. These results are similar to those reported by Ramadugu et al. (45).

*Haemophilus* is a genus of gram-negative bacteria, many of which can cause various illnesses and infections. The *Neisseria* genus contains several deadly or opportunistic bacterial pathogens but some species belong to commensal or nonpathogenic groups of oral microbiotas (46). Xu et al. (47) reported that the prevalence of *Rothia* was significantly reduced in caries-affected preschool children, suggesting that this genus is associated with dental health (47). Uranga et al. demonstrated increased interactions among *Rothia*, *Streptococcus*, and *Staphylococcus* species in responding to the biosynthesis of enterobactin by Rothia (48). An increase in the relative abundance of the genera *Rothia*, *Alloprevotella*, and *Haemophilus* has been associated with oral diseases and cancer (49, 50). Although all these bacterial groups can colonize mucosal surfaces, including the oral cavity, the current study revealed that they could be detected in infants' saliva. Questions regarding the significance of the initial acquisition and composition establishment in the oral environment remain unanswered.

Linear discriminant analysis effect size (LEfSe) was specifically designed for group comparisons of the detected changes in microbiome relative abundance between the groups (30). More precisely, LEfSe quantifies the magnitude of the associations between the microbial profiles. LEfSe analysis in this study showed that the abundances of at least 15 genera were significantly different between the mother-infant dyads based on a Kruskal–Wallis test. Interestingly, the *Staphylococcus* genus was more abundant in the early life of the infants (1.7% in the postpartum period) but was excluded from the microbial core in the saliva in infants 9 months after birth. The differences in relative abundance between mother-infant dyads diminished at 15 months.

Few studies have reported *Staphylococcus* colonization in infant saliva. As an important member of the skin microbiota, various *Staphylococci* species have been commonly isolated from the skin, saliva, bloodstream, and fecal samples of infants (51–53). Maternal breastfeeding practices and environmental exposure are considered infants' primary sources of infection. Another important finding of this study was that the differences in *Corynebacterium* abundance became less significant from postpartum to 15 months of age. It was also excluded from the microbial core in the infant's saliva 9 months after birth. It remains unclear how *Staphylococcus* and *Corynebacterium* genera play roles in the ecology of the normal oral microbiota in early human life and how they evolve and significantly influence one's future health and diseases. The findings of this study provide additional evidence that can be used to generate new hypotheses for future investigations.

Breast milk is an important source of various bacteria that can be transmitted to the oral cavity of infants. The correlation between human breast milk and the increased or decreased growth of *S. mutans* in infants' saliva has been controversial for decades (38, 54–56). Most previous studies have focused on the outcomes and associations between breastfeeding and early childhood caries. However, little is known about the association between breastfeeding and oral microbial diversity in infants. A cross-sectional study published by Holgerson et al. in 2013 demonstrated that the oral microbiota profiles of 3-month-old infants were significantly different between breastfed and formula-fed infants (57). A more recent study showed that the salivary microbial diversity of breastfed infants was substantially lower than that of non-breastfed infants at two months of age. Still, the differences diminished at 12 months (45). In our study, 32 healthy infants were examined, and only five were exclusively breastfed for 15 months. We observed differences in microbiome diversity and composition between breastfed and non-breastfed infants, notably a higher abundance of *Streptococcus* sp. and *Veillonella* sp. in breastfed infants and a higher abundance of *Rothia* sp. in non-breastfed infants. However, the overall differences were not statistically significant between the two groups. A possible explanation for these results could be the small sample size; therefore, these findings were preliminary. A growing body of evidence points to a dynamic relationship between the oral environment and the oral microbiota composition. Future studies with a larger sample size are required to investigate the impact of feeding practices on oral microbial acquisition and establishment in infants.

## 5. Conclusion and future research

The oral microbiota plays a significant role in human health (58). The early establishment of oral microbiota is essential in developing infants' immune systems and overall health. Here, we used a well-validated MiSeq sequencing platform, the HOMI*NGS* assay, and bioinformatics approaches to examine the profile of the oral microbiota of 32 healthy newborn infants from postpartum to 15 months of age, a critical period of microbial establishment in the first year of life in humans. Our findings demonstrate that (1) *Streptococcus* genus is the dominant component of the salivary microbiota in infants, with a relatively low prevalence of *S. mutans*. (2) The oral microbial composition was markedly different between mother-infant dyads during the first 15 months of infant's life. (3) The oral microbial relative abundance and diversity of the core oral microbiome increased with age for the infants but were relatively stable for the mothers. (4) The study did not find differences in microbial diversity between male and female infants and infants with different feeding experiences. Collectively, these findings enhance our understanding of the acquisition, subsequent development, and stability of a health-associated oral microbiome community in infants.

The current study was limited by the relatively small number of mother-infant dyads. Thirty-two mother-infant dyads were recruited during the postpartum visit, but only 13 pairs completed the follow-up visits. A high proportion of sequencing reads was unidentifiable at the microbial species level, limiting further exploration of microbial interactions. Therefore, our findings may not be generalized to other populations. In addition, detailed information about the oral health status, including caries experiences and periodontal health, of the mothers is not available. More maternal attributes ought to take into consideration for future studies to investigate the dynamics of oral microbiome acquisition in healthy infants and the potential long-term impact of the early establishment of the oral microbiome on the infant's overall health development.

## Data Availability

The raw reads generated in this study were deposited into the NCBI Sequence Read Archive (SRA) database under Bioproject PRJNA925651 and accession number SUB12552944. Questions regarding data access should be directed to yl3428@cornell.edu.
