# Supplementary material for: Dynamics of oral microbiome acquisition in healthy infants: A pilot study
Source: Front Oral Health. 2023 Mar 30;4:1152601. doi: 10.3389/froh.2023.1152601 (PMC10098328; doi:10.3389/froh.2023.1152601)
Supplement: Supplementary file 1 [file Datasheet1.docx]

**Dynamics of Oral Microbiome Acquisition in Healthy Infants: A Pilot Study**

Yihong Li^1*^, Prakaimuk Saraithong^2^, Lanxin Zhang^3^, Ashley Dills^4^, Bruce J. Paster^, 5,6^, Jin Xiao^7^, Tong Tong Wu^8^, Zachary Jones^9^

^1^ Master of Public Health Program, Department of Public and Ecosystem Health, Cornell University, Ithaca, NY 14853

^2^ Department of Internal Medicine, Medical School University of Michigan School of Dentistry, Ann Arbor, MI 48109

^3^ Department of Molecular and Cell Biology, University of California Berkeley, Oakland, CA 94709

^4^ Family Translational Research Group, New York University College of Dentistry, New York, NY 10010

^5^ Molecular Microbiology & Genetics, The Forsyth Institute, Cambridge, MA 02142

^6^ The Department of Oral Medicine, Infection, and Immunity, Harvard School of Dental Medicine, Boston, MA 02115

^7^ Eastman Institute for Oral Health, University of Rochester Medical Center, Rochester, NY 14642

^8^ Department of Biostatistics and Computational Biology, University of Rochester Medical Center, Rochester, NY 14642

^9^ Department of Basic Science and Craniofacial Biology, New York University College of Dentistry, New York, NY 10010

*** Correspondence:**Yihong Li, DDS, MPH, DrPH
Professor of Practice
Master of Public Health Program

Department of Public and Ecosystem Health
Cornell University,

S2012 Schurman Hall

Ithaca, NY 14853

Tel: [606-253-3467](tel:212-998-9607)

Email: [yl3428@cornell.edu](mailto:yl3428@cornell.edu)

**Running title:** Oral microbiota in infants and mothers

Keywords: Oral microbiota, microbial diversity, infant-mother dyads, postpartum, microbial initial acquisition

Supplementary Material

1. **Study sample size estimations**

The study sample size estimation was based on our previously published data on the early acquisition of *S. mutans* in infant saliva. Using the traditional culture methods, the cumulative positive rates for *S. mutans* were 7%-15%, 45%-58%, and 83%--85% at age 12, 24, and 36 months, respectively (Caufield et al., 1993; Li et al., 2005). The null hypothesis was that the initial *S. mutans* acquisition age was the same for healthy infants compared to previous studies. As shown in Table S1, 32 families provide at least 80% of statistical power to predict the difference in the cumulative positive rates for *S. mutans* colonization between the present study and previous studies.

**Supplementary Table S1.** The statistical power estimation to detect the initial *S. mutans* colonization in infants.

The second set of hypotheses was that the oral microbiome diversity in the infant saliva increases with age. The statistical power was estimated assuming that the predicted difference in the Shannon Index was 45% between the baseline [Shannon Index = 2.02] and 15 months of age [Shannon Index = 3.69] (Blostein et al., 2021; Wu et al., 2022). In our study, there are eight mother-infant pairs completed all three visits. As showed in Table S2 and Figure S1, eight mother-infant pairs who completed both postpartum and 15-month visit provides 88% statistical power for detecting a significant difference in the Shannon Index between visits. The power calculations were based on α = 0.05 type I error for a two-sided test.

**Supplementary Table 2.** The statistical power estimation for the microbiome study.

**Supplementary Figure S1.** Power estimations. The study has at least 80% of statistical power to detect the difference in microbial diversity measured by the Shannon Index in the infant’s saliva samples between different visits.


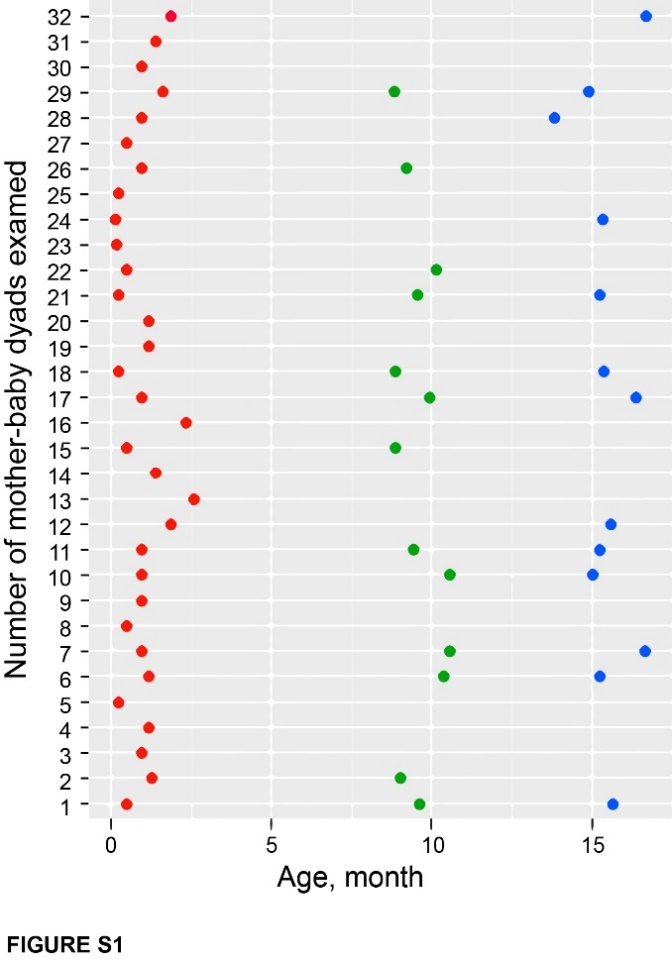


**Supplementary Figure S2.** The study population. At the baseline visit (postpartum in red), 32 mother-infant dyads participated in the study, 13 mother-infant dyads completed the 9-month visit (in green) and the 15-month visit (in blue).


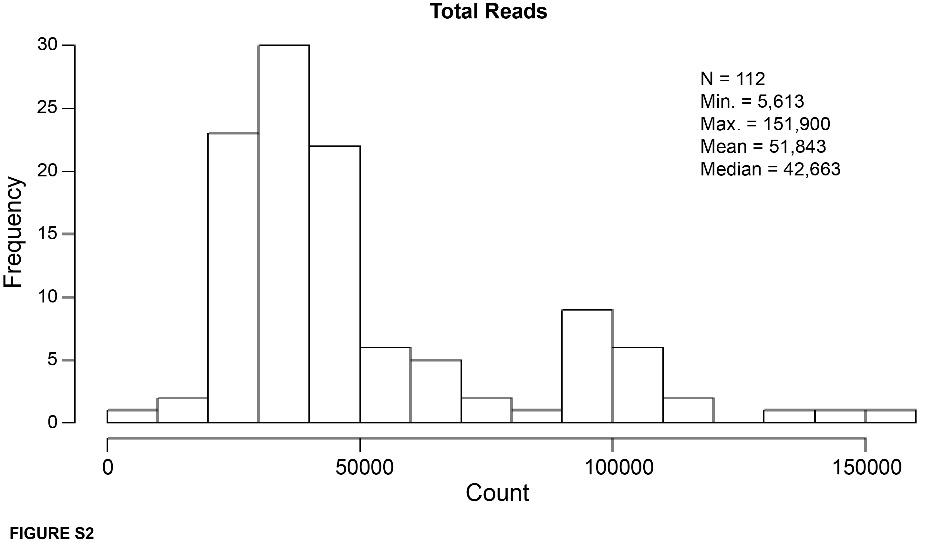


**Supplementary Figure S3.** The library size overview. We used 2000 as a cut-off that removes two extremely low counts samples (counts 118 and 1579). The majority of the samples have read counts above 12,000.


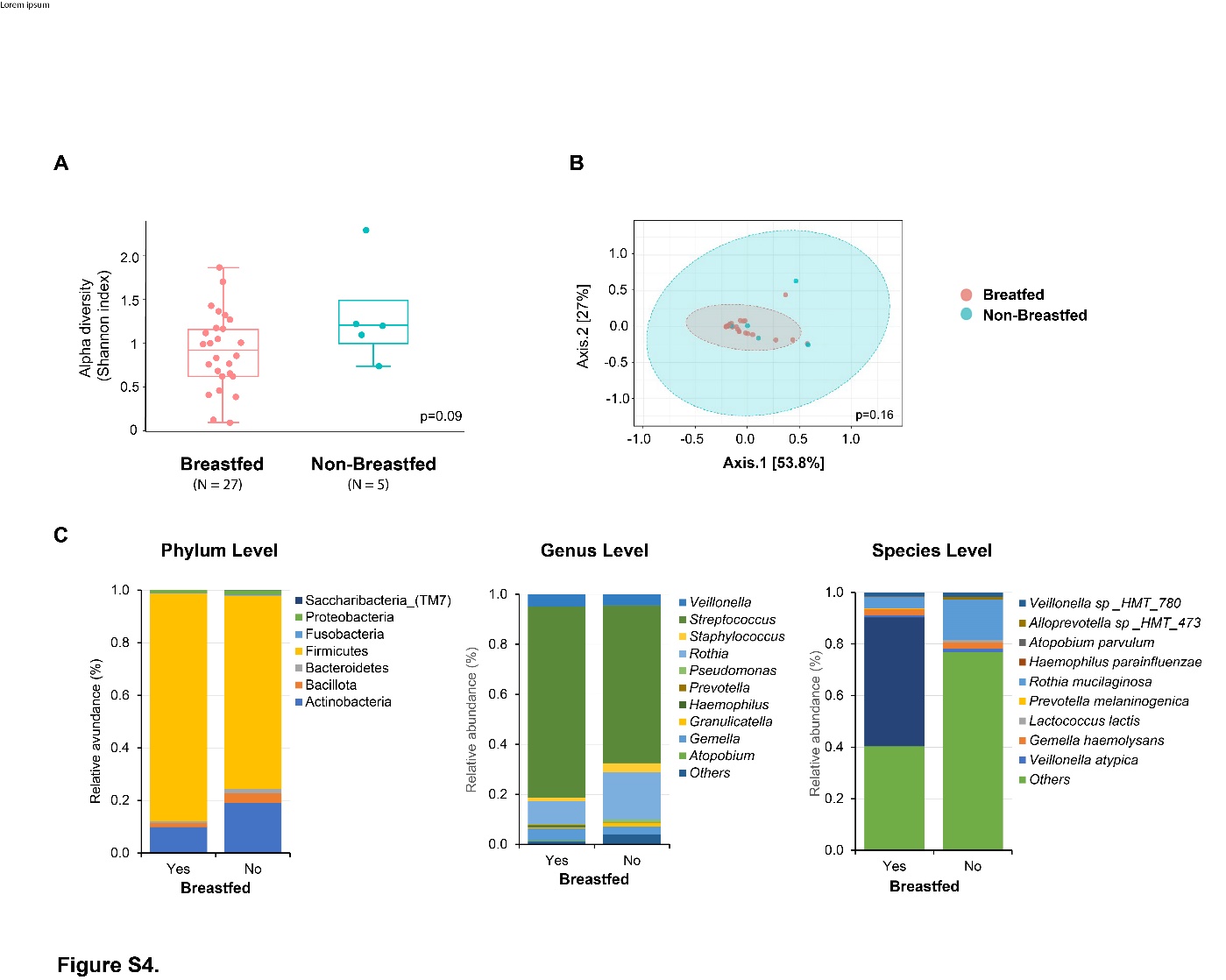


**Supplementary Figure S4.** Influence of feeding methods on the oral microbiome in neonates (1 month). **(A)** Alpha diversity Shannon Index and **(B)** Beta diversity Bray-Curtis Index of infants at postpartum. A comparison was made between breastfed and bottle-fed infants. **(C)** Relative abundance at phylum, genus, and species levels, showing 7 phyla and 10 most abundant genera and species. All graphs show that breastfeeding did not significantly impact the microbial communities in infants.


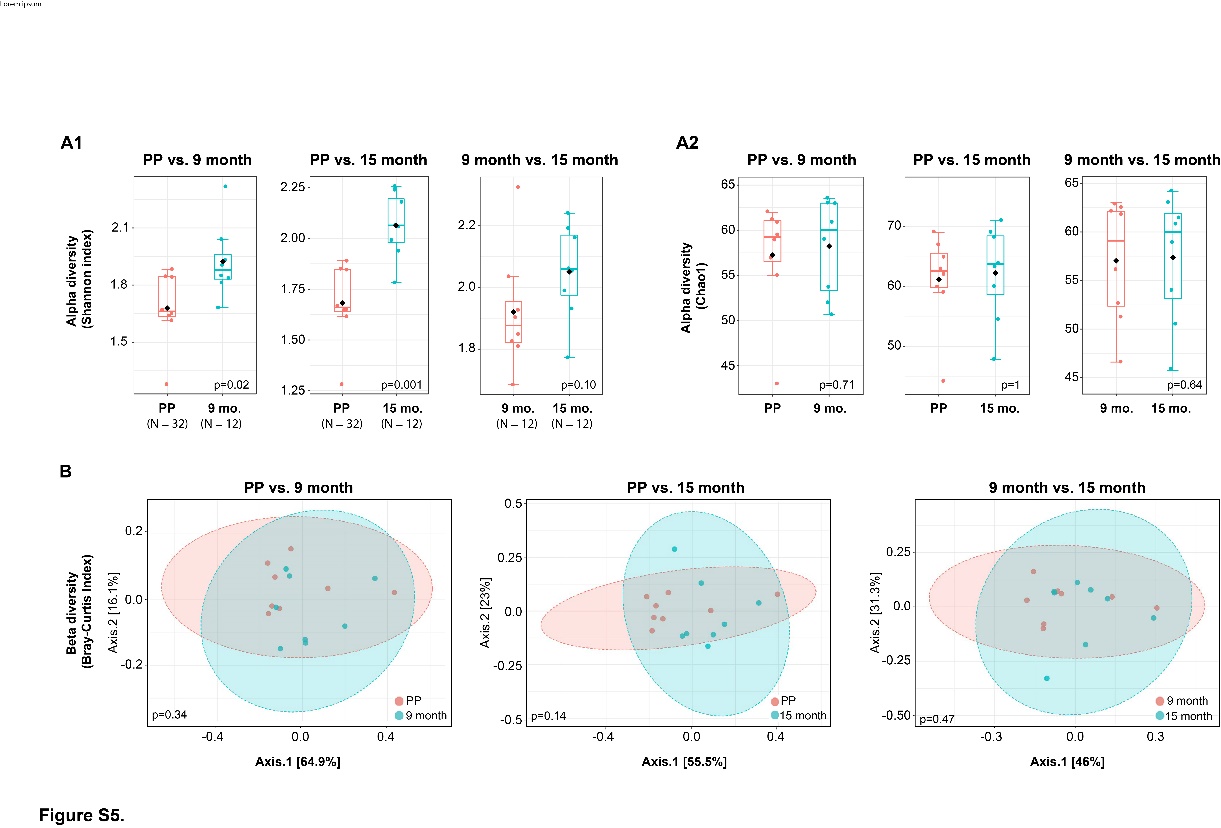


**Supplementary Figure S5. Stability of maternal oral microbiome during the postpartum period.** Only mothers who completed all three visits were included (N = 8). **(A1)** Shannon Index at the species level. **(A2)** Chao 1 at the species level. **(B)** Bray-Curtis Index at the species level. A2 and B show no significant difference at different time points. A1 demonstrates substantial changes from postpartum to 9 months and from postpartum to 15 months. However, no significant change is found from 9 months to 15 months.


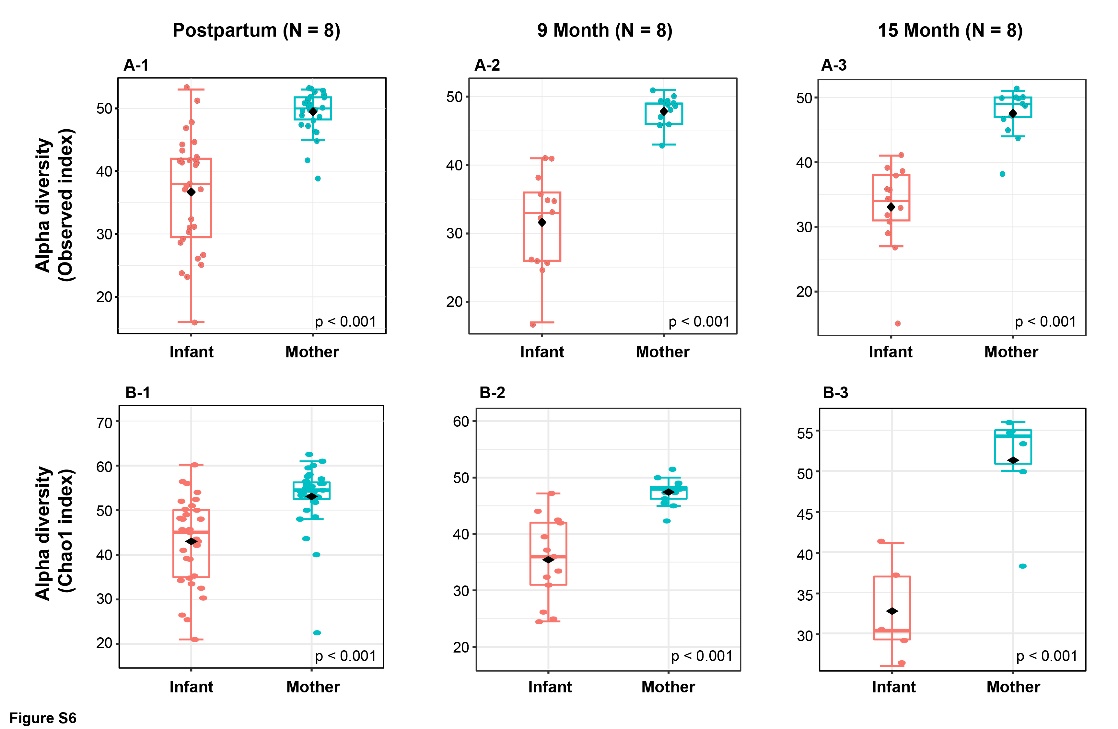


**Supplementary Figure S6.** Comparisons of alpha diversity between mother-infant paired samples. In the study, eight mother-infant pairs completed all three visits. (**A1-A3**) shows the alpha diversity measured by Observed Index. (**B1-B3**) shows the alpha diversity measured by Chao1. Significant differences were detected between mothers and infants at all three-time points (postpartum, 9 months, and 15 months) for both analyses, (p<0.001).

**Reference**

Blostein, F., Foote, S., Salzman, E., Mcneil, D.W., Marazita, M.L., Martin, E.T., and Foxman, B. (2021). Associations Between Salivary Bacteriome Diversity and Salivary Human Herpesvirus Detection in Early Childhood: A Prospective Cohort Study. *J Pediatric Infect Dis Soc* 10**,** 856-863.

Caufield, P.W., Cutter, G.R., and Dasanayake, A.P. (1993). Initial acquisition of mutans streptococci by infants: evidence for a discrete window of infectivity. *J Dent Res* 72**,** 37-45.

Li, Y., Caufield, P.W., Dasanayake, A.P., Wiener, H.W., and Vermund, S.H. (2005). Mode of delivery and other maternal factors influence the acquisition of *Streptococcus mutans* in infants. *J Dent Res* 84**,** 806-811.

Wu, T.T., Xiao, J., Manning, S., Saraithong, P., Pattanaporn, K., Paster, B.J., Chen, T., Vasani, S., Gilbert, C., Zeng, Y., and Li, Y. (2022). Multimodal data integration reveals mode of delivery and snack consumption outrank salivary microbiome in association with caries outcome in Thai children. *Front Cell Infect Microbiol* 12**,** 881899.
